# Nontuberculous Mycobacteria, Botswana, 2011–2014

**DOI:** 10.3201/eid2507.181440

**Published:** 2019-07

**Authors:** Bontle Mbeha, Madisa Mine, Modisa Sekhamo Motswaledi, John Dewar

**Affiliations:** National Tuberculosis Reference Laboratory, Gaborone, Botswana (B. Mbeha);; National Health Laboratory, Gaborone (M. Mine);; University of Botswana, Gaborone (M.S. Motswaledi);; University of South Africa, Pretoria, South Africa (J. Dewar)

**Keywords:** Botswana, nontuberculous mycobacteria, Mycobacterium avium-intracellulare, Tuberculosis and other mycobacteria, bacteria, drug susceptibility

## Abstract

We documented a 6-fold increase in the frequency of nontuberculous mycobacteria isolated from clinical samples in Botswana during 2011–2014. Because antituberculosis treatment is often initiated only on the basis of acid-fast bacilli smear-positive microscopy results, some patients with nontuberculous mycobacterial infections might have received inappropriate treatment.

Nontuberculous mycobacteria (NTM) isolated from clinical samples are often classified as contaminants (*1*). However, some NTM species are pathogenic to humans (*2*) and, in some parts of the world, cause more illness than infection with *Mycobacterium tuberculosis* does (*3*).

In Botswana, as in many other developing countries, patients with acid-fast bacilli–positive sputum are presumed to be infected with *M. tuberculosis* and are treated with antituberculosis agents (*4*), even though acid-fast bacilli smear microscopy does not distinguish between *M. tuberculosis* and NTM, and most antituberculosis drugs might not be effective against NTM (*5*). This observation, along with the number of increasing reports of NTM worldwide (*1*,*4*,*6*–*9*), prompted this study.

## The Study

During October 2015, we retrospectively analyzed 36,242 electronic records from 2011–2014 that were stored at the National Tuberculosis Reference Laboratory (Gaborone, Botswana) (Table). The records represented specimens referred for tuberculosis (TB) culture from 52 facilities across the country. Demographic parameters comprised age and sex. We compared proportions by calculating z values from observed frequencies, then used an online calculator to derive p values.

**Table:** Comparison of *Mycobacterium tuberculosis* and nontuberculous mycobacteria isolates, Botswana, 2011–2014*

Year	Total no. samples analyzed	*Mycobacterium tuberculosis*			Nontuberculous mycobacteria	
		No. (%) isolates	Incidence		No. (%) isolates	Incidence
2011	11,799	228 (1.9)	9.9		113 (0.96)	4.9
2012	6,357	526 (8.3)	22.9		370 (5.8)	16
2013	9,429	681 (7.2)	29.7		823 (8.7)	35.9
2014	8,657	594 (6.9)	25.9		693 (8.0)	30

*Per capita calculated on the basis of total country population. Incidence is per 100,000 population.

The proportions of *M. tuberculosis* and NTM cases during the study period were comparable (5.6% vs. 5.5%). This finding creates a high possibility of misdiagnosis and inappropriate treatment of *M. tuberculosis*/NTM cases. Most (74.5%) NTM samples were sputum; 22% were gastric aspirates, and the remaining 3.4% were obtained from other body sites. Of specimens from which NTM was isolated, 53.3% were from male patients and 43.4% were from female patients; for 3.1%, patient sex was not captured. The first specimen submitted per patient was analyzed. From the analysis we removed duplicate samples that might have resulted from submission of samples for follow-up from the same patient.

**Figure 1:**
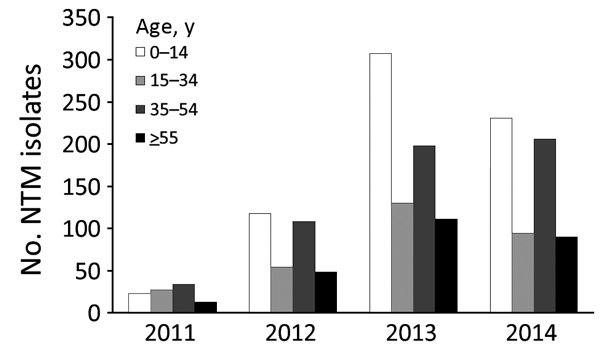
Age range of study population and total number of NTM isolates per year, Botswana, 2011–2014. NTM, nontuberculous mycobacteria.

One third (33.5%) of samples from which NTM was isolated were from patients 0–14 years of age, followed by 27.3% from patients 35–54 years of age (Figure 1). For at least 207 (10.4%) of the 1,999 NTM samples, the age of the patient was not captured.

**Figure 2:**
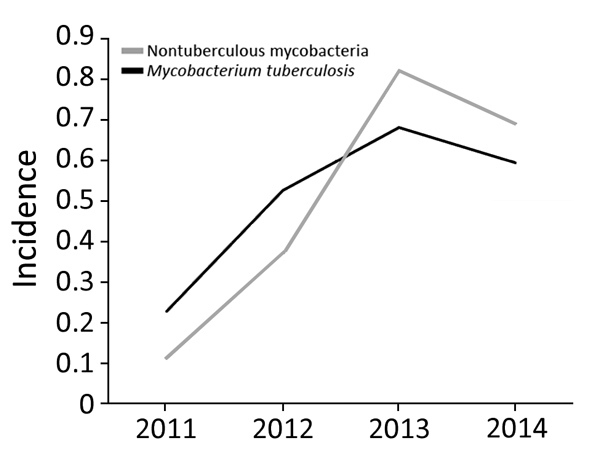
Incidence per 100,000 population of nontuberculous mycobacteria and *Mycobacteria tuberculosis* isolates in clinical specimens, Botswana, 2011–2014. For both, p<0.0001.

Overall, the proportion of isolated NTM increased 6-fold, from 0.113/100,000 population in 2011 to 0.693/100,000 in 2014 (the estimated Botswana population in 2017 was 2.292 million). Moreover, isolation of NTM significantly increased during 3 periods: 2011–2012, 2012–2013, and 2011–2014 (p<0.0001 for each period). We found no significant change in NTM occurrence for 2013–2014 (Figure 2).

Before 2013, NTM was not speciated in Botswana. In 2013, a total of 324 (39.4%) of 823 NTM isolates were speciated. Of these 324 samples, 161 (49.7%) were *M. intracellulare*, the NTM species most frequently isolated, followed by *M. malmoense* (17 [5.2%] of 324) and *M. gordonae* (15 [4.6%] of 324). Seventy-eight (24.0%) isolates could be identified only as *Mycobacterium* spp. because of a limitation of the assay used. Seventy-four (46.0%) patients in whom *M. intracellulare* was isolated were also HIV positive, suggesting that NTM might be an important opportunistic pathogen in HIV infection. *M. avium-intracellulare* has also been reported to cause most pulmonary infections in several studies (*8*,*10*,*11*). These reports are useful because failure to speciate NTM isolates or conduct drug susceptibility testing might be overlooking an emerging pathogen with potential to cause illness and even death in HIV-infected persons.

Most samples were from male patients, a finding consistent with findings by Moore et al. (*1*). This finding has been attributed to risk factors such as increased rates of smoking and chronic obstructive pulmonary disease among men (*1*). However, investigators in the United States observed a female predominance (*3*).

## Conclusions

We observed a high occurrence of NTM in patients 35–54 years of age. We also noted an unexpected increase of >30% in reported NTM cases in children <14 years of age, which might represent a peculiar childhood vulnerability to NTM infections.

According to the Botswana National Tuberculosis Programme Manual, no information is available in Botswana about the prevalence of disseminated NTM (*12*). Our study provides basic but crucial data that may stimulate discussion toward policy change in the laboratory investigation and treatment of NTM isolates for better patient care.

The change in 2011 in the use of media from Lowenstein-Jensen to MGIT (Beckton-Dickinson, https://www.bd.com) culture might have contributed to an increase in NTM identification. A similar study by Chihota et al. (*13*) yielded more NTM after such a change. However, if the increase in this study resulted from a change in media, only the first year would have recorded an increase, followed by a stabilizing trend in subsequent years. Therefore, the increasing trend we observed reflects a convincing climb in NTM cases. Furthermore, our results suggest that patients with NTM might have received unnecessary TB treatment (*7*). This finding underscores the need to unequivocally identify mycobacterial isolates because NTM appears to be more frequently encountered (*9*).

Our study has limitations. Our results might not reflect the prevalence of NTM in the country because the samples analyzed comprised only persons suspected to have TB. This fact notwithstanding, the National Tuberculosis Referral Laboratory is the sole laboratory in Botswana that performs culture and thus represents a reasonable sampling of NTM countrywide among persons suspected to have TB infection. The prevalence of NTM might therefore be higher than that reported in this study.

We propose that mycobacterial isolates be routinely speciated and subjected to drug susceptibility testing. We base this proposal on our observation that NTM is isolated at nearly the same frequency as *M. tuberculosis* and is isolated predominantly in children <14 years of age. Treatment of NTM as *M. tuberculosis* underestimates the clinical significance of NTM and is likely to negatively affect treatment outcomes and inflate reports of *M. tuberculosis*.
